# Application of a Waveguide-Mode Sensor to Blood Testing for Hepatitis B Virus, Hepatitis C Virus, Human Immunodeficiency Virus and *Treponema pallidum* Infection

**DOI:** 10.3390/s19071729

**Published:** 2019-04-11

**Authors:** Shigeyuki Uno, Takenori Shimizu, Torahiko Tanaka, Hiroki Ashiba, Makoto Fujimaki, Mutsuo Tanaka, Koichi Awazu, Makoto Makishima

**Affiliations:** 1 Division of Biochemistry, Department of Biomedical Sciences, Nihon University School of Medicine, Itabashi-ku, Tokyo 173-8610, Japan; uno.shigeyuki@nihon-u.ac.jp (S.U.); shimizu.takenori16@gmail.com (T.S.); torahiko.tanaka@gmail.com (T.T.); 2 Electronics and Photonics Research Institute, National Institute of Advanced Industrial Science and Technology (AIST), 1-1-1 Higashi, Tsukuba, Ibaraki 305-8565, Japan; h.ashiba@aist.go.jp (H.A.); m-fujimaki@aist.go.jp (M.F.); k.awazu@aist.go.jp (K.A.); 3 Department of Life Science & Green Chemistry, Saitama Institute of Technology, 1690 Fusaiji, Fukaya, Saitama 369-0293, Japan; mutsuo-tanaka@sit.ac.jp

**Keywords:** waveguide-mode sensor, on-site blood testing, hepatitis B virus, hepatitis C virus, human immunodeficiency virus, *Treponema pallidum*

## Abstract

Testing for blood-transmitted infectious agents is an important aspect of safe medical treatment. During emergencies, such as significant earthquakes, many patients need surgical treatment and/or blood transfusion. Because a waveguide mode (WM) sensor can be used as a portable, on-site blood testing device in emergency settings, we have previously developed WM sensors for detection of antibodies against hepatitis B virus and hepatitis C virus and for forward ABO and Rh(D) and reverse ABO blood typing. In this study, we compared signal enhancement methods using secondary antibodies conjugated with peroxidase, a fluorescent dye, and gold nanoparticles, and found that the peroxidase reaction method offers superior sensitivity while gold nanoparticles provide the most rapid detection of anti-HBs antibody. Next, we examined whether we could apply a WM sensor with signal enhancement with peroxidase or gold nanoparticles to detection of antibodies against hepatitis C virus, human immunodeficiency virus and *Treponema pallidum*, and HBs antigen in plasma. We showed that a WM sensor can detect significant signals of these infectious agents within 30 min. Therefore, a portable device utilizing a WM sensor can be used for on-site blood testing of infectious agents in emergency settings.

## 1. Introduction

Blood tests for infectious agents, such as hepatitis B virus (HBV), hepatitis C virus (HCV), human immunodeficiency virus (HIV) and *Treponema pallidum* (TP), are required before surgical treatment and blood transfusion in order to protect patients and medical personnel from infection [1,2]. Although screening tests for infectious agents are routinely performed at major hospitals and blood examination centers, laboratories cannot be expected to work effectively during an emergency or natural calamity due to building collapse, disrupted electrical power supply and damage or injury to laboratories and personnel [3]. During large-scale incidents, such as significant earthquakes, many victims need surgical treatment and/or blood transfusion. Therefore, the availability of on-site examination for infectious agents as well as blood typing will be helpful in providing life-saving care.

A waveguide-mode (WM) sensor is a portable device designed to detect molecules and particles on a sensor chip utilizing electric field enhancement, a technology similar to surface plasmon resonance (SPR) sensor [4,5]. A WM sensor uses waveguide modes, instead of SPR [4,6]. A WM sensor chip is composed of a layer of silicon sandwiched with a SiO_2_ layer and a SiO_2_ substrate. The wavelength of a WM sensor is widely controllable over the visible light spectrum by adjusting the thickness of the top SiO_2_ and embedded silicon layers. Although the material used to induce SPR restricts the wavelength of incident light for a SPR sensor, this restriction is not applicable to a WM sensor. We have developed WM sensors for detection of antigen-antibody complexes and successfully applied the method to forward ABO and Rh(D) blood typing, reverse ABO blood typing, and detection of antibodies against HBV and HCV in human plasma [7,8,9,10]. We can determine ABO and Rh(D) blood typing with WM sensors utilizing hemagglutination detection within 3.5 min [7,8,9]. An enhancing method using a peroxidase reaction increases sensitivity for detection of antibodies against HBV and HCV requires additional processing time of about 90 min [10]. Although signal enhancement using colored nanoparticles or gold nanoparticles increases detection sensitivity in a WM sensor as well as in a SPR sensor [11,12,13,14,15], these methods have not been studied in blood testing by a WM sensor. In this study, we developed a WM sensor-based method to detect antigen-antibody complexes in plasma samples more quickly utilizing signal enhancement methods and applied the method to the detection of anti-HCV antibody, anti-HIV antibody, anti-TP antibody and HBV surface antigen.

## 2. Materials and Methods

### 2.1. WM Sensors

**Figure 1:**
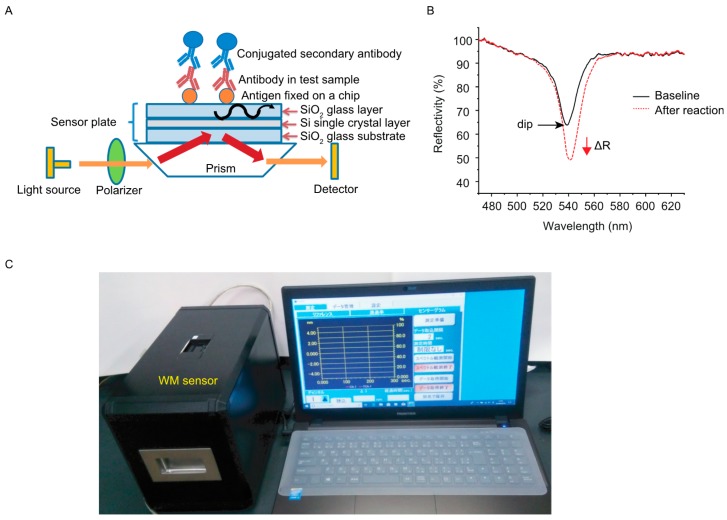
A waveguide mode (WM) sensor. (**A**) Schematic diagram of a WM sensor for detection of antigen-antibody complexes. A sensing chip is composed of 3 layers made of SiO_2_ and Si. For detection of antibody in test sample, antigen is fixed on a sensor chip. Secondary antibody is conjugated with a fluorescent dye, gold nanoparticles or HRP for signal enhancement. (**B**) Illustration of dips in reflectance spectra. Interaction of colored target molecules on a chip changes a dip shape as ΔR in depth. (**C**) A WM sensor apparatus.

Optical arrangement of a WM sensor device was based on the Kretschmann configuration as reported previously [7,8,10,11]. Briefly, when polarized light enters through a prism at a particular angle of incidence, light of a specific wavelength is propagated in a slab waveguide of a sensing chip, a phenomenon known as excitation of the WM, which causes decrease in the reflectance of light. When target molecules are captured on a sensing chip, the interaction influences the reflective property of the waveguide surface and the complex refractive index near the waveguide surface can be evaluated as a change in the depth and/or wavelength of the dip in reflectance spectra (Figure 1A). While the interaction of colorless target molecules on a chip preferentially induces a shift of the wavelength of the dip peak, the presence of colored target molecules induces a change in the dip depth (ΔR) [4,16] (Figure 1B). When the wavelength of WM overlaps that of conjugated materials or dye, a change in the depth of the dip in reflectance spectra becomes larger [4,11]. A prism was made of SiO_2_ glass, the bottom angle of the prism was 38°. A sensor chip was composed of a SiO_2_ glass substrate, an embedded silicon layer and an upper SiO_2_ layer. The thicknesses of the embedded silicon and upper SiO_2_ layers was adjusted for resonance wavelength of 530 nm [9]. We examined several fluorescent dyes and gold nanoparticles for signal enhancement in preliminary experiments, and selected HiLyte Fluor 555 (absorbance wavelength: 555 nm) and 80 nm gold nanoparticles (absorbance wavelength: 553 nm). Horseradish peroxidase (HRP) catalyzes the conversion of 3-amino-9-ethylcarbazole (AEC) to red water-insoluble precipitates, which induce a larger change in the dip depth [10]. All measurements were performed at room temperature with a WM sensor device EVA-001 (Optex, Otsu, Japan) (Figure 1C).

### 2.2. Antigens and Antibodies

HBs adr antigen and HCV NS3, NS4 and NS5 antigens were purchased from ProSpec (East Brunswick, NJ, USA), HCV core antigen and treponemal Tp15, Tp17 and Tp47 antigens were from Abcam (Cambridge, United Kingdom), HIV gp36 and gp41 antigens were from LKT laboratories (St. Paul, MN), mouse monoclonal anti-HBs antibodies (Hs33 and Hs41) were from HyTest (Turku, Finland), and rabbit polyclonal anti-HBs antibody was from Beacle (Kyoto, Japan).

### 2.3. Fixation of Antigens and Antibodies on Sensor Chips

A SiO_2_ surface of a sensor chip was chemically modified with triethoxysilane derivatives, C12Es (silane bearing succinimide ester moiety) and M3EG (silane bearing methoxytriethylene glycol moiety) [17]. The modified chips, which have *N*-hydroxysuccinimide ester residues reactive with free amino acid residues, were incubated with 20 μg/ml antibody or antigen for 12 h, washed with water, and placed in phosphate buffered saline (PBS).

### 2.4. Detection of Mouse Monoclonal Anti-HBs Antibody with a WM Sensor

PBS was placed on a chip fixed with HBs adr antigen at room temperature, a baseline spectrum was measured. To compare signal enhancement methods, mouse anti-HBs antibody (Hs41) in PBS (50 µL) was added for an antigen-antibody reaction on a chip for 10 min and the chip surface was washed with PBS 5 times. Fluorescence- and gold nanoparticle-conjugated antibodies were prepared using anti-mouse IgG antibody reacted with the HiLyte Fluor 555 Labeling kit–NH_2_ (Dojindo Laboratories, Kumamoto, Japan) and the 80 nm NHS-activated Gold Nanoparticle Conjugation kit (Cytodiagnostics, Burlington, Canada), respectively. Fluorescence-conjugated antibody (50 µL; 10-fold dilution) or gold nanoparticle-conjugated antibody (50 µL; 15-fold dilution) was incubated on a chip for 5 min. After washing with PBS five times, changes in the spectrum dip were measured with a WM sensor. The signal enhancement method using a peroxidase reaction was also performed with EnVision+System–HRP (anti-mouse) (antibody conjugated with HRP; Dako North America, Carpinteria, CA) as reported previously with a minor modification [10]. Peroxidase-conjugated antibody (no dilution) was incubated on a chip for 5 min. After washing with PBS 5 times, the peroxidase reaction was employed with AEC solution (Vector Laboratories, Burlingame, CA) for 1 min. Changes in the spectrum dip were measured with a WM sensor.

### 2.5. Detection of Antibodies against HCV, HIV and TP in Human Plasma with a WM Sensor

Human plasma containing antibody for HCV, HIV or TP were obtained from SeraCare (Milford, MA), and control plasma were obtained from healthy volunteers with approval of the Research Ethics Committee and Clinical Research Judging Committee of Nihon University School of Medicine (reference number 25-6-1). HCV core, NS3, NS4 and NS5 antigens, HIV gp36 and gp41 antigens, or TP Tp15, Tp17 and Tp47 antigens were fixed on a sensor chip, and a baseline spectrum was measured. Test plasma (50 µL) were subjected to an antigen-antibody reaction on sensor chip for 10 min, and the chip surface was washed with PBS 5 times. For signal enhancement with gold nanoparticles, 80 nm gold nanoparticle-conjugated anti-human IgG antibody (50 µL; 15-fold dilution) was added, and changes in the spectrum dip were measured with a WM sensor. For signal enhancement with a peroxidase reaction, MAHG-SZ (Anti-human IgG [H, γ-chain specific]-Strongzyme HRP Conjugate, Mouse-Mono; Stereospecific Detection Technologies, Baesweiler, Germany) (50 µL; 20,000-fold dilution) was added for 5 min. After washing with PBS 5 times, AEC solution was added and changes in the spectrum dip were measured with a WM sensor.

### 2.6. Detection of HBs Antigen in Human Plasma with a WM Sensor

HBs antigen-positive human plasma (SeraCare) and control plasma from healthy volunteers were utilized as antibody detection methods. Anti-HBs monoclonal antibody (Hs33) was fixed on a sensor chip, and a baseline spectrum was measured. Test plasma (50 µL) were subjected to an antigen-antibody reaction on a chip for 10 min, and the chip surface was washed with PBS 5 times. For signal enhancement with gold nanoparticles, 80 nm gold nanoparticle-conjugated rabbit anti-HBs antibody (50 µL; 15-fold dilution) was added, and changes in the spectrum dip were measured with a WM sensor. For signal enhancement with a peroxidase reaction, rabbit anti-HBs polyclonal antibody was added for 5 min. After washing with PBS 5 times, EnVision+System–HRP (anti-rabbit) (Dako) (no dilution) was incubated for 5 min. After further washing with PBS 5 times, AEC solution was added and changes in the spectrum dip were measured with a WM sensor.

### 2.7. Statistical Analysis

Data are presented as means ± S.D. We performed the two-tailed, unpaired Student’s *t* test to assess significant differences between the two groups using Origin 2019 (OriginLab Corporation, Northampton, MA). A *p* value < 0.05 was considered to be statistically significant.

## 3. Results

### 3.1. Detection of Mouse Anti-HBs Antibody with a WM Sensor and Signal Enhancement Methods

**Figure 2:**
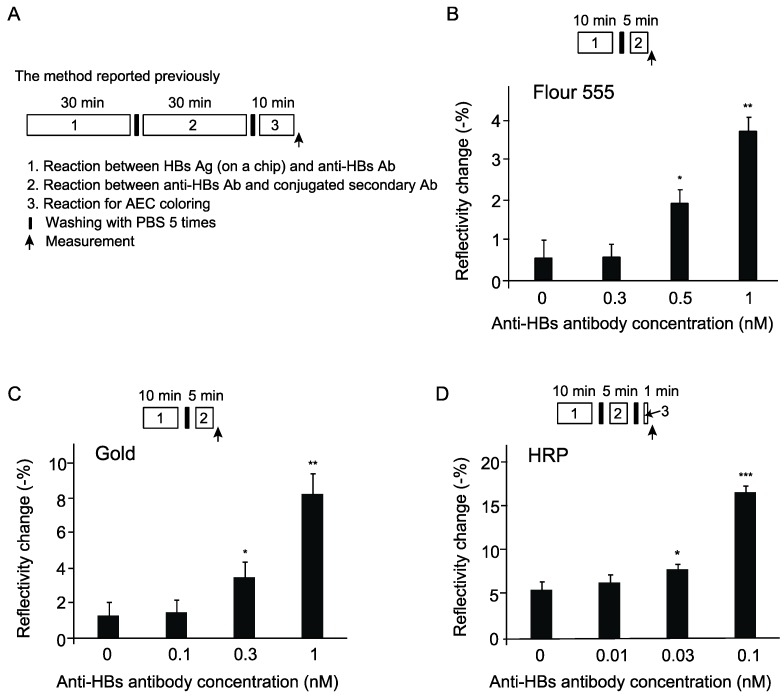
Comparison of signal enhancement methods in detection of anti-HBs antibody with a WM sensor. (**A**) Experimental procedure of the previous method using a peroxidase reaction [10]. Ag, antigen. Ab, antibody. (**B**) Signal enhancement using secondary antibody conjugated with the fluorescent dye (Fluor 555). (**C**) Signal enhancement using gold nanoparticle-conjugated secondary antibody. (**D**) Time shortened method using HRP-conjugated secondary antibody. * *p* < 0.05, ** *p* < 0.01, *** *p* < 0.001 versus control sample without anti-HBs antibody (n = 3 for each group).

In our previous study, we successfully increased the detection sensitivity for anti-HBs antibody with a WM sensor utilizing a peroxidase reaction with AEC [10]. This method takes about 90 min in total, including 30 min to allow interaction between HBs antigen on a chip and anti-HBs antibody, 30 min for interaction between anti-HBs antibody and peroxidase-conjugated secondary antibody, 10 min for an enzymatic reaction of peroxidase on AEC, and repeated washing between each reaction (Figure 2A). In order to shorten process time, we examined non-enzymatic enhancement methods using fluorescence-conjugated antibody or gold nanoparticle-conjugated antibody instead of peroxidase-conjugated antibody. We shortened the reaction time for interaction between HBs antigen and anti-HBs antibody to 10 min and could detect anti-HBs antibody at 0.5 nM and 0.3 nM using fluorescence-conjugated antibody and gold nanoparticle-conjugated antibody, respectively, within 20 min (Figure 2B,C). The signal enhancement using gold nanoparticle-conjugated antibody is more sensitive than that using fluorescence-conjugated antibody in a WM sensor, although it’s less sensitive than the previous method using a peroxidase reaction, which can detect 0.03 nM anti-HBs antibody [10]. Next, we examined whether we can shorten process time of a peroxidase reaction enhancement method without decreasing detection sensitivity. Incubation time for an interaction between HBs antigen and anti-HBs antibody was decreased from 30 min to 10 min, incubation time for anti-HBs antibody and peroxidase-conjugated antibody was decreased from 30 min to 5 min, and peroxidase reaction time was shortened from 10 min to 1 min. In these experimental conditions, we detected 0.03 nM anti-HBs antibody within 20 min after the addition of anti-HBs antibody (Figure 2D). Since the detection limits of signal enhancement methods using peroxidase, the fluorescent dye, and gold nanoparticles were 0.03 nM, 0.5 nM, and 0.3 nM, respectively, for mouse anti-HBs antibody detection, the peroxidase reaction method was superior in sensitivity, although it needs more time than the methods using the fluorescent dye and gold nanoparticles. These results indicate that a WM sensor with a signal enhancement method can be used for rapid detection of an antigen-antibody reaction.

### 3.2. Detection of Antibodies Against HCV, HIV and TP in Plasma with a WM Sensor

**Figure 3:**
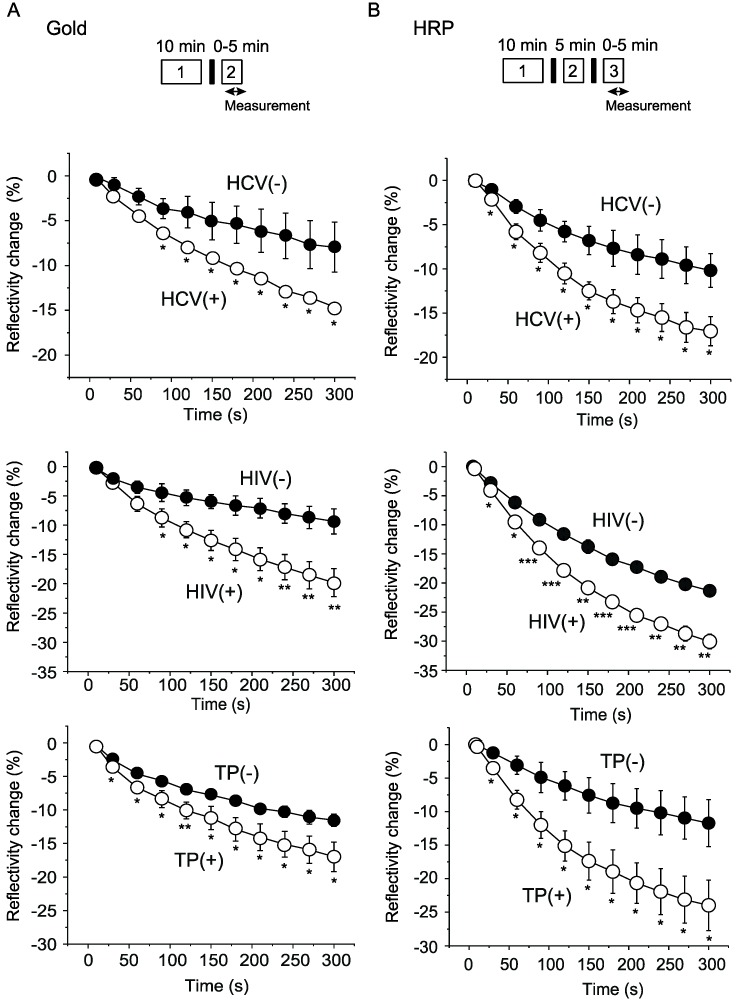
Detection of antibodies against HCV, HIV and TP with a WM sensor. (**A**) Signal enhancement using gold nanoparticle-conjugated antibody. Experimental procedure: 1, reaction between antigens fixed on a chip and antibodies against HCV, HIV or TP; 2, reaction between antibody against HCV, HIV or TP and conjugated secondary antibody. (**B**) Signal enhancement using HRP-conjugated antibody. Experimental procedure: 1 and 2 as (**A**); 3, reaction for AEC coloring. * *p* < 0.05, ** *p* < 0.01 versus control plasma (n = 3–4 for each group).

Previously, we successfully detected anti-HBs antibody in human blood samples with a WM sensor using signal enhancement [10]. We examined whether we can apply a WM sensor to detection of antibodies against HCV, HIV and TP in plasma. We obtained commercially available plasma positive for antibodies to HCV, HIV or TP, utilized a signal enhancement method with gold nanoparticle-conjugated antibody or peroxidase-conjugated antibody, and obtained time courses to assess change in a dip of a spectrum. The method using gold nanoparticle-conjugated antibody detected significant signal changes for antibodies against HCV, HIV and TP at time points of 60 s, 60 s and 30 s, respectively (Figure 3A). The method using a peroxidase reaction detected signal changes of AEC coloring specific for antibodies against HCV, HIV and TP at 30 s (Figure 3B). These findings indicate that we can detect antibodies against HCV, HIV and TP with WM sensors with gold nanoparticles and with peroxidase reaction with total process time of 11–12 and 18 min, respectively.

**Figure 4:**
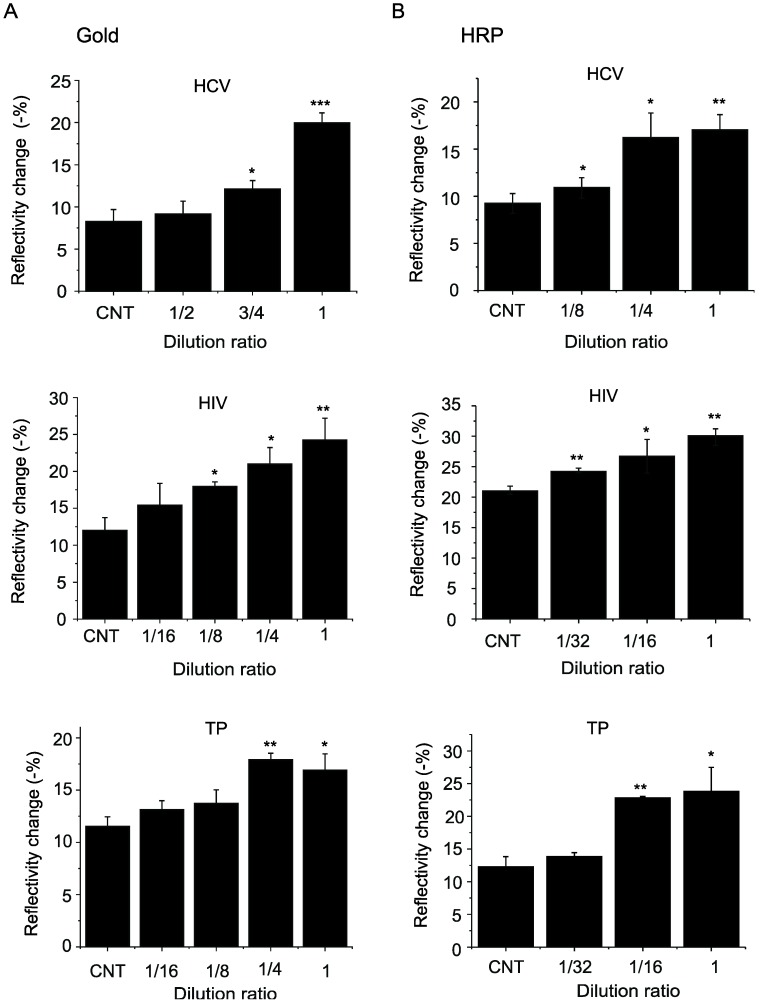
Comparison of detection sensitivity of signal enhancement using gold nanoparticles (**A**) and a peroxidase reaction (**B**). Changes in a dip of a spectrum were measured at the time point of 300 s (5 min) as shown in Figure 2. CNT, control plasma. Plasma positive for antibody against HCV, HIV or TP were diluted in indicated ratios. * *p* < 0.05, ** *p* < 0.01, *** *p* < 0.001 versus control plasma (n = 3 for each group).

We compared detection sensitivity of the gold nanoparticle and peroxidase reaction methods by diluting test plasma samples. The gold nanoparticle assay detected significant signal at 3/4 dilution, 1/8 dilution and 1/4 dilution of plasma positive for antibodies against HCV, HIV and TP, respectively (Figure 4A). The method using a peroxidase reaction was more sensitive, detecting significant signal at 1/8 dilution, 1/32 dilution and 1/16 dilution of plasma positive for antibodies against HCV, HIV and TP, respectively (Figure 4B).

### 3.3. Detection of HBs Antigen in Human Plasma with a WM Sensor

**Figure 5:**
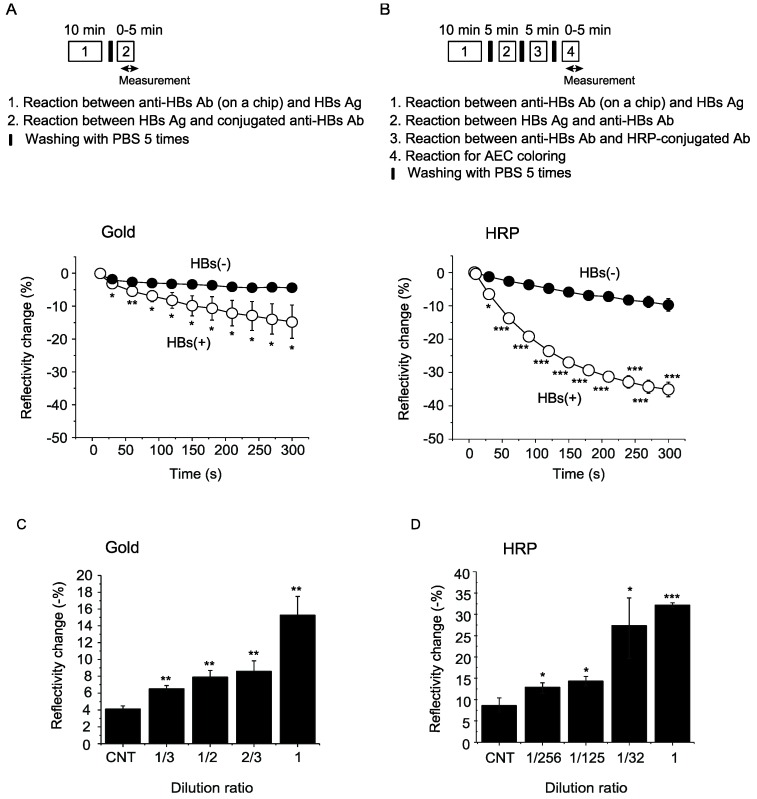
Detection of HBs antigen with a WM sensor. (**A**) Signal enhancement using gold nanoparticle-conjugated antibody. (**B**) Signal enhancement using HRP-conjugated antibody. Comparison of detection sensitivity of signal enhancement using gold nanoparticles (**C**) and a peroxidase reaction (**D**). Changes in a dip of a spectrum were measured at the time point of 5 min (300 s). CNT, control plasma. Plasma positive for HBs antigen were diluted in indicated ratios. * *p* < 0.05, ** *p* < 0.01, *** *p* < 0.001 versus control plasma (n = 3–4 for each group).

Detection of HBs antigen, together with testing for antibodies against HBV, HCV, HIV and TP, is used for blood screening before blood transfusion and surgery in Japan [1]. We examined whether we can detect HBs antigen in human plasma with a WM sensor. We utilized commercially available HBs antigen-positive plasma and compared signal enhancement methods using gold nanoparticles and peroxidase to detect HBs antigen in a WM sensor. We detected significant changes in dip of spectrum for HBs antigen using gold nanoparticle-conjugated anti-HBs antibody (Figure 5A). Because peroxidase-conjugated anti-HBs antibody was not commercially available, we used sequential treatment of HBs antigen with rabbit anti-HBs antibody and next with anti-rabbit peroxidase-conjugated antibody, and also detected significant changes in a dip of a spectrum for HBs antigen (Figure 5B). A method with peroxidase detected significant HBs-specific signals in 1/256 diluted plasma, while that with gold nanoparticle detected signal in 1/3 diluted plasma (Figure 5C,D). These findings indicate that a WM sensor can be used for detection of HBs antigen and that signal enhancement with a peroxidase reaction is more sensitive than that with gold nanoparticles.

## 4. Discussion

We have previously developed WM sensors for forward ABO and Rh(D) and reverse ABO blood typing and for detection of antibodies against HBV and HCV in plasma [7,8,9,10]. In the current study, we successfully detected antibodies against HIV and TP and HBs antigen in plasma with a WM sensor. We compared three signal enhancement methods using conjugation of secondary antibody with a fluorescent dye, gold nanoparticles and peroxidase and found that methods using gold nanoparticles and a peroxidase reaction are superior in rapidity and sensitivity, respectively (Figure 2). The detection limits of signal enhancement methods using gold nanoparticles and peroxidase were 0.3 nM and 0.03 nM, respectively for mouse anti-HBs antibody detection. In detection of human anti-HBV antibody by a SPR biosensor, the detection limits of a direct assay (without signal enhancement) and of signal enhancement methods using secondary antibody and peroxidase-anti-peroxidase complex were reported to be 9.20 nM, 4.39 nM, and 0.64 nM, respectively [18]. Although different antigen-antibody reactions were used for analysis, these findings suggest that a WM sensor is as sensitive as or more sensitive than a SPR sensor. A method using gold nanoparticle-conjugated secondary antibody can detect signals of anti-microbial antibodies in 11–12 min. A highly sensitive method using a peroxidase reaction needs more time but can detect signals in 18 min. Since detection of HBs antigen in plasma requires reaction with anti-HBs antibody and sequential reaction with peroxidase-conjugated secondary antibody, it takes about 25 min in total. We have developed a portable, small WM sensor with a battery power source and with microfluidic multi-channel chips (Ashiba and Awazu, unpublished). We have also developed a novel method for blood plasma separation without using centrifugation and successfully detected antibody against HBV in whole blood using a WM sensor equipped with a microfluidic channel combined with the plasma separation technique [19]. Therefore, a WM sensor with multichannel microfluidic chips can be used in emergency settings as a rapid on-site test instrument for blood screening.

We fixed sensor chips with commercially available HBs, HCV, HIV and TP antigens to detect antibodies against HBV, HCV, HIV and TP, respectively, and also used commercially available anti-HBs antibody to detect HBs antigen (Figure 3, Figure 4 and Figure 5). Recent immunological methods for anti-microbial antibody testing and for HBs antigen, such as chemiluminescent enzyme immunoassay, have developed by optimizing fixed proteins (antigens for antibody detection and antibodies for antigen detection) to enhance efficiency of the antigen-antibody reaction [20,21,22,23]. Detection sensitivity of WM sensor methods will be further increased by utilizing fixed proteins that are more specific and sensitive for antigen-antibody reactions. Nucleic acid amplification testing for infectious agents is a very useful tool at major hospitals and blood examination centers because of high sensitivity and high specificity [1,23]. However, the complexity of machinery for this detection method would no adapt well to the emergency setting [3]. Therefore, a WM sensor could be used as an on-site portable diagnostic device in emergency settings.

## 5. Conclusions

A portable device utilizing a WM sensor can be applied for the detection of anti-HCV antibodies, anti-HIV antibodies, anti-TP antibodies and HBV surface antigen in human plasma. Since a WM sensor chip has higher stability and versatility than a SPR sensor surface and the detection sensitivity of a WM sensor is comparable to or higher than that of a SPR sensor, a WM sensor will be useful for immunoassays. Further studies are needed to develop a WM sensor with multichannel microfluidic chips for on-site blood testing of infectious agents and blood typing.
